# The value on SUV-derived parameters assessed on ^18^F-FDG PET/CT for predicting mediastinal lymph node metastasis in non-small cell lung cancer

**DOI:** 10.1186/s12880-023-01004-7

**Published:** 2023-04-05

**Authors:** Xuhe Liao, Meng Liu, Shanshi Li, Weiming Huang, Cuiyan Guo, Jia Liu, Yan Xiong, Jianhua Zhang, Yan Fan, Rongfu Wang

**Affiliations:** 1 Department of Nuclear Medicine, Peking University First Hospital, No. 8, Xishiku St., West District, Beijing, 100034 China; 2 Department of Radiation Oncology, Peking University First Hospital, Beijing, 100034 China; 3 Department of Thoracic Surgery, Peking University First Hospital, Beijing, 100034 China; 4 Department of Respiratory and Critical Care Medicine, Peking University First Hospital, Beijing, 100034 China; 5 Department of Radiology, Peking University First Hospital, Beijing, 100034 China; 6 Department of Pathology, Peking University First Hospital, Beijing, 100034 China; 7 Department of Nuclear Medicine, Peking University International Hospital, No 1, Life Science Park, Zhongguancun, Changping District, Beijing, 102206 China

**Keywords:** NSCLC, Mediastinal lymph node metastasis, ^18^F-FDG, SUV, SUVpeak, Tumor marker

## Abstract

**Purpose:**

To explore valuable predictors for mediastinal lymph node metastasis in non-small cell lung cancer (NSCLC) patients, we analyzed the potential roles of standardized uptake value (SUV)-derived parameters from preoperative ^18^F-FDG PET/CT combined with clinical characteristics.

**Methods:**

Data from 224 NSCLC patients who underwent preoperative ^18^F-FDG PET/CT scans in our hospital were collected. Then, a series of clinical parameters including SUV-derived features [SUVmax of mediastinal lymph node and primary-tumor SUVmax, SUVpeak, SUVmean, metabolic tumor volume (MTV) and total lesion glycolysis (TLG)] were evaluated. The best possible cutoff points for all measuring parameters were calculated using receiver operating characteristic curve (ROC) analysis. Predictive analyses were performed using a Logistic regression model to determine the predictive factors for mediastinal lymph node metastasis in NSCLC and lung adenocarcinoma patients. After multivariate model construction, data of another 100 NSCLC patients were recorded. Then, 224 patients and 100 patients were enrolled to validate the predictive model by the area under the receiver operating characteristic curve (AUC).

**Results:**

The mediastinal lymph node metastasis rates in 224 patients for model construction and 100 patients for model validation were 24.1% (54/224) and 25% (25/100), respectively. It was found that SUVmax of mediastinal lymph node ≥ 2.49, primary-tumor SUVmax ≥ 4.11, primary-tumor SUVpeak ≥ 2.92, primary-tumor SUVmean ≥ 2.39, primary-tumor MTV ≥ 30.88 cm^3^, and primary-tumor TLG ≥ 83.53 were more prone to mediastinal lymph node metastasis through univariate logistic regression analyses. The multivariate logistic regression analyses showed that the SUVmax of mediastinal lymph nodes (≥ 2.49: OR 7.215, 95% CI 3.326–15.649), primary-tumor SUVpeak (≥ 2.92: OR 5.717, 95% CI 2.094–15.605), CEA (≥ 3.94 ng/ml: OR 2.467, 95% CI 1.182–5.149), and SCC (< 1.15 ng/ml: OR 4.795, 95% CI 2.019–11.388) were independent predictive factors for lymph node metastasis in the mediastinum. It was found that SUVmax of the mediastinal lymph node (≥ 2.49: OR 8.067, 95% CI 3.193–20.383), primary-tumor SUVpeak (≥ 2.92: OR 9.219, 95% CI 3.096–27.452), and CA19-9 (≥ 16.6 U/ml: OR 3.750, 95% CI 1.485–9.470) were significant predictive factors for mediastinal lymph node metastasis in lung adenocarcinoma patients. The AUCs for the predictive value of the NSCLC multivariate model through internal and external validation were 0.833 (95% CI 0.769- 0.896) and 0.811 (95% CI 0.712–0.911), respectively.

**Conclusion:**

High SUV-derived parameters (SUVmax of mediastinal lymph node and primary-tumor SUVmax, SUVpeak, SUVmean, MTV and TLG) might provide varying degrees of predictive value for mediastinal lymph node metastasis in NSCLC patients. In particular, the SUVmax of mediastinal lymph nodes and primary-tumor SUVpeak could be independently and significantly associated with mediastinal lymph node metastasis in NSCLC and lung adenocarcinoma patients. Internal and external validation confirmed that the pretherapeutic SUVmax of the mediastinal lymph node and primary-tumor SUVpeak combined with serum CEA and SCC can effectively predict mediastinal lymph node metastasis of NSCLC patients.

**Supplementary Information:**

The online version contains supplementary material available at 10.1186/s12880-023-01004-7.

## Introduction

In China, lung cancer ranks first in new cancer cases and cancer deaths, with 815,600 and 714,700 cases, respectively [1]. According to the WHO classification of lung cancer, the main pathological type of lung cancer is non-small cell lung cancer (NSCLC), accounting for 80% of all types of lung cancer [2].

Mediastinal lymph node metastasis is an important basis for the treatment decision of NSCLC [3]. ^18^F-FDG PET/CT examination has been widely practiced in clinics and plays a vital role in tumor staging, prognosis evaluation, efficacy monitoring and so on [3–6]. ^18^F-FDG PET/CT can evaluate the glucose metabolism of tumor tissue noninvasively. The N-staging value of ^18^F-FDG PET/CT in NSCLC patients has been recommended in international guidelines [3]. However, ^18^F-FDG is not a tumor-specific imaging agent, and its uptake can be influenced by tumoral molecular pathological characteristics (such as glucose transporter expression) and biological behaviors (such as the degree of differentiation). In addition, there could be high uptake of ^18^F-FDG in a variety of benign diseases (inflammation, tuberculosis, hyperplasia, etc.), which may affect the accuracy of diagnosis [5, 6]. Therefore, as far as patients with confirmed or highly suspicious lung cancer are concerned, the qualitative judgment of mediastinal lymph nodes requires not only quantitative evaluation of the glucose metabolism level of the lesion but also comprehensive analysis and evaluation combined with multiple clinical and pathological information of the patient, which is a collaborative process of multidisciplinary teams (MDTs) [7]. The maximum standardized uptake value (SUVmax) is the most common semiquantitative parameter of glucose metabolism imaging and has been accepted and recognized clinically as a malignant tumor evaluation index [7–10]. With the development of image processing technology and application of software, novel semiquantitative parameters have been applied, such as peak standardized uptake value (SUVpeak), average standardized uptake value (SUVmean), metabolic tumor volume (MTV) and total lesion glycolysis (TLG). Compared with SUVmax, these semiquantitative parameters can more comprehensively evaluate the glucose metabolism level of the lesion [5, 6, 9, 10], and these parameters derived from standardized uptake can be collectively referred to as SUVs, which have been proven to be valuable in the prediction of mediastinal lymph node metastasis [7, 11, 12]. Nevertheless, some studies have also reported that although SUVs could improve the judgment of mediastinal lymph node metastasis, different clinical studies reported different diagnostic cutoff values of SUVs [7–10], and their prediction efficiencies were greatly affected by the subject population, country of study origin, scanner makes and other factors, and each center should formulate corresponding standards based on the situation of the center [7]. Furthermore, potential factors related to mediastinal lymph node metastasis also include patient age, maximum diameter of primary tumor, serum tumor markers, solid degree of primary tumor, pathological subtype, etc. [7, 11, 12].

Therefore, based on the data of our center in recent years, we combined ^18^F-FDG PET/CT semiquantitative parameters and a series of clinicopathological information to carry out a retrospective analysis of the prediction of mediastinal lymph node metastasis in NSCLC.

## Materials and methods

### Patients

The retrospective data collection and analysis procedures were approved by the Institutional Review Board (IRB) of our hospital, which waived the need for written informed consent.

Data from 324 newly diagnosed NSCLC patients who underwent ^18^F-FDG PET/CT scans at our hospital were retrospectively collected. Based on variables of the model from 224 NSCLC patients during the period of December 2011 to April 2020, corresponding data of another 100 NSCLC patients were recorded from May 2020 to January 2021.

The inclusion criteria were as follows: (1) diagnosed NSCLC was made by primary tumor pathological analyses, including cytological and histological evaluations and *EGFR* gene evaluations; (2) resection of pulmonary primary tumor and dissection of mediastinal lymph node had been completed after ^18^F-FDG PET/CT scans; (3) ^18^F-FDG PET/CT scans and blood tests were performed before treatments, and *EGFR* mutation and *ALK* rearrangement analysis preceded other therapies except surgery; and (4) the time interval between surgery and examinations did not exceed four weeks.

The exclusion criteria were as follows: patients with a history of malignancy in five years.

### General data collection

Individual information of 224 NSCLC patients was recorded, including (1) demographic characteristics: age, sex, smoking status; and (2) clinical parameters relevant to EGFR gene status: TNM stage and serum tumor markers (CEA, CYFRA21-1, CA19-9, SCC, NSE, TPA and proGRP). Then, based on variables of the model from 224 patients, corresponding data of another 100 NSCLC patients were recorded.

The TNM stage was determined according to the 8th edition of the International Association for the Study of Lung Cancer (IASLC) TNM staging system.

### PET/CT acquisition and analysis

PET/CT scanning was implemented on a Philips Gemini GXL 16 PET/CT. Sixty to eighty minutes after the intravenous injection of ^18^F-FDG (3.7 MBq/kg body weight), patients were imaged from the skull base to the mid-thigh level in the supine position by unenhanced low-dose CT and PET. The reconstructed images were fused by EBW V3.5.2.2264 and PET/CT Application Suite V1.5.1A software. The SUV-derived parameters were acquired by MedEx MEMRS V8.0 software.

All images of PET/CT scans were independently evaluated by 3 board-certified nuclear medicine physicians (ML, JHZ, and YF with 20 y, 22 y and 25 y of experience in interpreting oncologic PET/CT, respectively, which is ∼ 800 scans/y) in a blinded manner. The final judgment result was determined by the majority principle. Tumor burden was calculated with three-dimensional volumes of interest (VOIs) drawn on the volume of metabolic tumor-related activity by applying a percentage threshold of 40% [13]. SUVs included SUVmax, SUVpeak, SUVmean, MTV and TLG originating from the primary tumor and SUVmax of the mediastinal lymph node. SUVmax was defined as tissue radioactivity concentration/(injected dose/patient weight). SUVmean was defined as the average SUV related to the tumor burden. MTV, defined as lesion volume with uptake, was calculated within the tumor burden, and TLG was calculated as SUVmean multiplied by MTV. The maximum diameter of the primary tumor was analyzed and determined in noncontrast breath-hold CT simultaneously. The maximum diameter of the primary focus was defined as the maximum diameter of the solid component of the tumor. The degree of solidity was divided into partial solid and solid. To ensure the accuracy of SUVmax determination of mediastinal lymph nodes, mediastinal lymph nodes of all patients in this study were measured in a nonblind method. For the patients with mediastinal lymph node metastasis verified by postoperative pathology, the highest SUVmax of mediastinal lymph nodes was selected among the metastatic mediastinal lymph nodes as the index of SUVmax of mediastinal lymph nodes. The matching process of imageologic and pathologic lymph nodes was completed though the cooperation of pathologists and nuclear medicine physicians. For patients without metastasis in mediastinal lymph nodes proven by postoperative pathology, the highest SUVmax of all mediastinal lymph nodes was selected as the SUVmax of the mediastinal lymph node.

### Pathological analysis

Tissue specimens of primary tumors were acquired through biopsy or surgical resection. Pathological analysis was conducted by pathologists at the Department of Pathology of our hospital.

All the patients in our study received hilar and mediastinal lymph node dissection, and these lymph nodes were confirmed as positive or negative through histopathological analysis. The pathological report of lymph nodes includes the number of lymph nodes dissected in each area and the number of positive lymph nodes in each area. Mediastinal lymph node zoning was based on the American Thoracic Society map of regional pulmonary nodes in 2009.

*EGFR* mutations were tested using immunohistochemical (IHC) analysis or quantitative polymerase chain reaction (qPCR). In brief, for IHC, EGFR mutation-specific antibodies were Rabbit XP® mAbs obtained from Cell Signaling Technology. For the qPCR method, amplification was performed using the AmoyDx™ Human EGFR Mutation Detection Kit. The amplification was set up, and the final run files were interpreted according to the manual of the manufacturer by the ABI 7500 Real-time PCR system. ALK mutation was detected by IHC [ventana ALK (d5f3) CDX assay from Roche Diagnostics usa] or PCR [eml4-alk fusion gene detection kit from Amoy Diagnostics Co., Ltd., China] [9].

### Statistical analysis

Other CT and PET/CT images and clinical, pathologic, and genetic information were performed blinded to predict outcome data.

All parameters were compared according to the metastatic status in mediastinal lymph nodes using the chi-squared test and Student’s t-test/Mann–Whitney test. Receiver operating characteristic (ROC) curves were constructed to obtain the cutoff value of continuous variables.

All parameters with *p* < 0.05 in the univariate logistic analysis were further analyzed by multivariate regression analysis. Variates with *p* < 0.05 in the multivariate analysis were deemed independent predictors for metastasis in mediastinal lymph nodes, and the odds ratios and 95% confidence intervals (CI) of the predictors were obtained.

The area under the curve (AUC) was used to evaluate the predictive value of the constructed model. All *p* values < 0.05 were considered significant. All analyses were performed using the SPSS 25.0 software package.

**Fig. 1:**
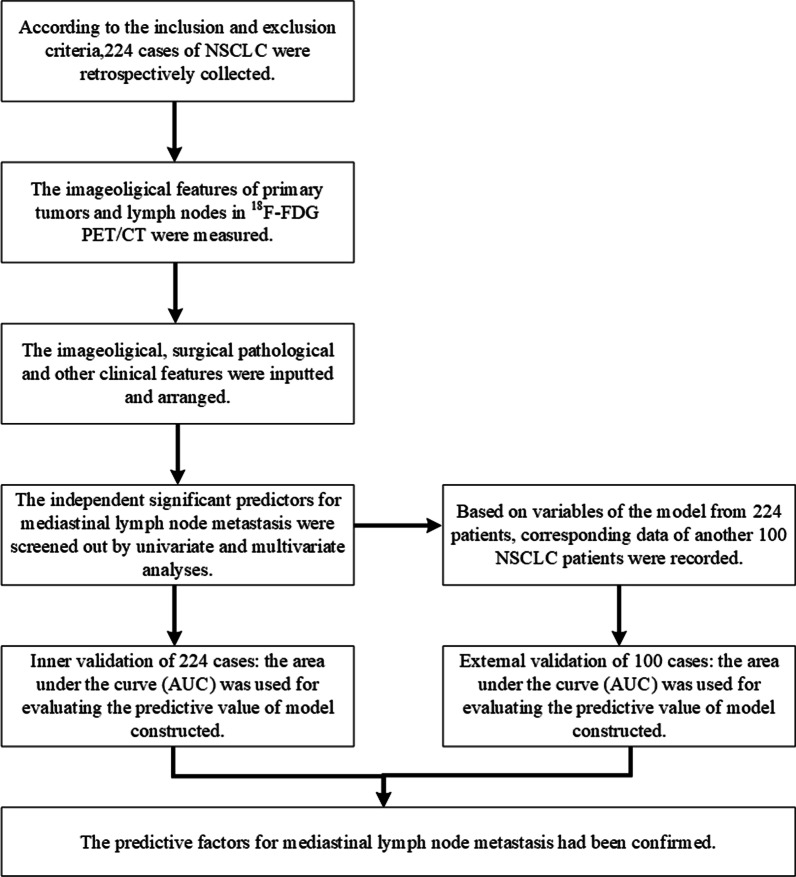
The process of this study

The process of this study is displayed in Fig. 1.

## Results

### General characteristics

A total of 224 patients with newly diagnosed NSCLC were enrolled in model construction in this study, including 137 men (61.2%) and 87 women (38.8%) with an age of 63.48 ± 9.57 years (36–83 years) [mean ± standard deviation (range)]. Mediastinal lymph node metastasis occurred in 54 cases (54/224, 24.1%). There were 162 cases of lung adenocarcinoma (162/224, 72.3%) and 54 cases of lung squamous cell carcinoma (54/224, 24.1%). The rates of mediastinal lymph node metastasis in lung adenocarcinoma and squamous cell carcinoma were 25.3% (41/162) and 16.7% (9/54), respectively. (Table 1).

**Table 1:** Basic information of NSCLC patients for model construction and model validation

Characteristics	Model construction (N = 224)	Model validation (N = 100)	Value	*p* value
Age	63.48 ± 9.57	63.33 ± 9.54	− 0.128^a^	0.898
*Gender*			0.692^**b**^	0.406
Male	137	66		
Female	87	34		
*Smoking history*			0.481^**b**^	0.488
No	128	53		
Yes	96	47		
SUVmax of mediastinal lymph node	1.87 (1.41, 3.29)	2.03 (1.41, 3.76)	11,625.00^**d**^	0.585
Primary-tumor SUVmax	7.13 (3.05, 11.28)^**c**^	7.46 (2.78, 11.91)	11,572.50^**d**^	0.632
Primary-tumor SUVpeak	4.48 (2.11, 7.33)^**c**^	5.14 (2.00, 8.31)	11,546.00^**d**^	0.657
Primary-tumor SUVmean	4.20 (1.78, 6.82)^**c**^	4.35 (1.57, 7.15)	11,528.00^**d**^	0.647
Primary-tumor MTV	16.51 (9.14, 35.75)^**c**^	15.73 (9.14, 37.65)	11,164.50^**d**^	0.964
Primary-tumor TLG	53.99 (19.15, 228.81)^**c**^	57.43 (19.65, 244.25)	11,313.00^**d**^	0.885
Primary tumor size (mm)	28.00 (20.00, 40.00)^**c**^	27.50 (20.00, 38.00)	11,350.00^**d**^	0.847
CEA	3.24 (1.95, 5.63)^**c**^	3.07 (1.98, 5.12)	10,935.00^**d**^	0.734
CYFRA21-1	2.83 (2.10, 4.14)^**c**^	2.66 (2.07, 3.96)	10,731.50^**d**^	0.547
CA19-9	11.22 (8.21, 17.30)^**c**^	10.47 (7.26, 17.26)	10,465.00^**d**^	0.345
SCC	0.93 (0.70, 1.40)^**c**^	1.00 (0.73, 1.40)	11,502.50^**d**^	0.697
NSE	13.05 (11.31, 15.63)^**c**^	12.89 (10.79, 15.60)	10,691.50^**d**^	0.514
TPA	87.56 (63.19, 134.98)^**c**^	82.26 (63.15, 129.53)	10,948.50^**d**^	0.747
proGRP	43.55 (34.30, 54.16)^**c**^	44.87 (35.64, 55.24)	11,837.50^**d**^	0.413
*EGFR* mutation			0.589^**b**^	0.443
No	166	70		
Yes	58	30		
*ALK* rearrangement			0.238^**b**^	0.626
No	205	95		
Yes	14	5		

*NSCLC*, non-small cell lung cancer; *SUV*, standardized uptake value; *SUVmax*, maximum SUV; *SUVpeak*, peak of SUV; *SUVmean*, average of SUV; *MTV*, metabolic tumor volume; *TLG*, total lesion glycolysis; *FDG*, fluorodeoxyglucose; *CEA*, carcinoembryonic antigen; *CYFRA21-1*, cytokeratin 19 fragment; *CA19-9*, carbohydrate antigen 19–9; *SCC*, squamous cell carcinoma antigen; *NSE*, neuron-specific enolase; *TPA*, tissue polypeptide antigen; *proGRP*, precursor of gastrin releasing peptide; *EGFR*, epidermal growth factor receptor; *ALK*, anaplastic lymphoma kinase

^a^T test. ^b^Pearson Chi-Square test. ^c^Median [Q1, Q1)]. ^d^Mann-Whiteney test

One hundred patients with newly diagnosed NSCLC were collected as validation data for model construction, including 66 men (66.0%) and 34 women (34.0%) with an age of 63.33 ± 9.54 years (38–83 years). Mediastinal lymph node metastasis occurred in 25 cases (25/100, 25.0%). Among them, there were 71 cases of lung adenocarcinoma (71.0%) and 29 cases of lung squamous cell carcinoma. The rates of mediastinal lymph node metastasis in lung adenocarcinoma and squamous cell carcinoma were 25.4% (18/71) and 24.1% (7/29), respectively.

TNM staging: According to operative pathological analysis, there were 116 patients with stage I (89 Ia and 27 Ib), 28 with stage II (3 IIa and 25 IIb), 63 with stage III (45 IIIa, 13 IIIb and 5 IIIc), and 17 with stage IV (14 IVa and 3 IVb) in 224 patients; there were 49 patients with stage I (37 Ia and 12 Ib), 9 with stage II (1 IIa and 8 IIb), 30 with stage III (10 IIIa, 8 IIIb and 2 IIIc), and 12 with stage IV (10 IVa and 2 IVb) in 100 patients.

### Analysis of the correlation of SUV-derived parameters and clinical characteristics with metastasis in mediastinal lymph nodes

As shown in Table 2 and Additional file 1: Table S1, metastases in mediastinal lymph nodes were found more frequently in high SUVmax of mediastinal lymph node (≥ 2.49), high SUVmax of primary tumor (≥ 4.11), high SUVpeak of primary tumor (≥ 2.92), high SUVmean of primary tumor (≥ 2.39), high MTV of primary tumor (≥ 30.88 cm^3^), high TLG of primary tumor (≥ 83.53), large maximum diameter of primary tumor (≥ 32.5 mm), solid primary tumor, high CEA (≥ 3.94 ng/ml), high CYFRA21-1 (≥ 2.10 ng/ml), high CA19-9 (≥ 16.6 U/ml), low SCC (< 1.15 ng/ml) and high NSE (≥ 14.66 ng/ml).

**Table 2:** Association between main clinical variables and situation of mediastinal lymph nodes in 224 NSCLC patients

Variable	Mediastinal lymph node metastasis		Total	Value^a^	*p* value
	No	Yes			
*SUVmax of mediastinal lymph node*				36.378	< 0.001
< 2.49	132 (88.0%)	18 (12.0%)	150 (67%)		
≥ 2.49	38 (51.4%)	36 (48.6%)	74 (33%)		
*Primary-tumor SUVmax*				16.525	< 0.001
< 4.11	70 (92.1%)	6 (7.9%)	76 (33.9%)		
≥ 4.11	100 (67.6%)	48 (32.4%)	148 (66.1%)		
*Primary-tumor SUVpeak*				18.187	< 0.001
< 2.92	73 (92.4%)	6 (7.6%)	79 (35.3%)		
≥ 2.92	97 (69.9%)	48 (33.1%)	145 (64.7%)		
*Primary-tumor SUVmean*				14.430	< 0.001
< 2.39	66 (91.7%)	6 (8.3%)	72 (32.1%)		
≥ 2.39	104 (68.4%)	48 (31.6%)	152 (67.9%)		
*Primary-tumor MTV (cm*^*3*^*)*				7.066	0.008
< 30.88	132 (80.5%)	32 (19.5%)	164 (73.2%)		
≥ 30.88	38 (63.3%)	22 (36.7%)	60 (26.8%))		
*Primary-tumor TLG*				10.243	0.001
< 83.53	111 (83.5%)	22 (16.5%)	133 (59.4%)		
≥ 83.53	59 (64.8%)	32 (35.2%)	91 (40.6%)		
*Primary solid tumor maximum diameter (mm)*				10.243	0.001
< 32.50	111 (83.5%)	22 (16.5%)	133 (59.4%)		
≥ 32.50	59 (64.8%)	32 (35.2%)	91 (40.6%)		
*Degree of primary-tumor solidity*				17.705	< 0.001
Partial solid	76 (91.6%)	6 (8.4%)	83 (37.1%)		
Solid	94 (66.7%)	47 (33.3%)	141 (62.9%)		
*CEA (ng/ml)*				15.043	< 0.001
< 3.94	119 (84.4%)	22 (15.6%)	141 (62.9%)		
≥ 3.94	51 (61.4%)	32 (38.6%)	83 (37.1%)		
*CYFRA21-1 (ng/ml)*				5.16	0.023
< 2.10	48 (87.3%)	7 (12.7%)	55 (24.6%)		
≥ 2.10	122 (72.2%)	47 (27.8%)	169 (75.4%)		
*CA19-9 (U/ml)*				6.065	0.014
< 16.6	130 (80.2%)	32 (19.8%)	162 (72.3%)		
≥ 16.6	40 (64.5%)	22 (35.5%)	62 (27.7%)		
*SCC (ng/ml)*				4.199 ^a^	0.04
< 1.15	103 (71.5%)	41 (28.5%)	144 (64.3%)		
≥ 1.15	67 (83.7%)	13 (16.3%)	80 (35.7%)		
*NSE (ng/ml)*				4.552	0.033
< 14.66	121 (80.1%)	30 (19.9%)	151 (67.4%)		
≥ 14.66	49 (67.1%)	24 (32.9%)	73 (32.6%)		

*NSCLC*, non-small cell lung cancer; *SUV*, standardized uptake value; *SUVmax*, maximum SUV; *SUVpeak*, peak of SUV; *SUVmean*, average of SUV; *MTV*, metabolic tumor volume; *TLG*, total lesion glycolysis; *CEA*, carcinoembryonic antigen; *CYFRA21-1*, cytokeratin 19 fragment; *CA19-9*, carbohydrate antigen 19-9; *SCC*, squamous cell carcinoma antigen; *NSE*, neuron-specific enolase

^a^Pearson chi-square test

### Predictive factor analysis for metastasis in mediastinal lymph nodes

In univariate logistic regression analysis, SUVmax of mediastinal lymph node, SUVmax of primary tumor, SUVpeak of primary tumor, SUVmean of primary tumor, MTV of primary tumor, TLG of primary tumor, maximum diameter of primary tumor, degree of primary-tumor solidity, CEA, CYFRA21-1, CA19-9, SCC and NSE were significantly related to metastasis in mediastinal lymph nodes (Table 3 and Additional file 1: Table S2).

**Table 3:** Univariate and multivariate Logistic regression analysis of predictive factors for mediastinal lymph node in NSCLC patients

Characteristics	Univariate analysis OR (95% CI)	*p* value	Multivariate regression OR (95% CI)	*p* value
*SUVmax of mediastinal lymph node*				
< 2.49	Reference		Reference	
≥ 2.49	6.947 (3.551–13.591)	< 0.001	7.215 (3.326–15.649)	< 0.001
*Primary-tumor SUVmax*				
< 4.11	Reference			
≥ 4.11	5.600 (2.272–13.801)	< 0.001		
*Primary-tumor SUVpeak*				
< 2.92	Reference		Reference	
≥ 2.92	6.021 (2.444–14.829)	< 0.001	5.717 (2.094–15.605)	0.001
*Primary-tumor SUVmean*				
< 2.39	Reference			
≥ 2.39	5.077 (2.058–12.525)	< 0.001		
*Primary-tumor MTV (cm*^*3*^*)*				
< 30.88	Reference			
≥ 30.88	2.388 (1.244–4.583)	0.009 ^b^		
*Primary-tumor TLG*				
< 83.53	Reference			
≥ 83.53	2.737 (1.460–5.128)	< 0.002		
*Degree of primary-tumor solidity*				
Partial solid	Reference			
solid	5.429 (2.321–12.697)	< 0.001		
*CEA (ng/ml)*				
< 3.94	Reference		Reference	
≥ 3.94	3.394 (1.800–6.400)	< 0.001	2.467 (1.182–5.149)	0.016
*CYFRA21-1 (ng/ml)*				
< 2.10	Reference			
≥ 2.10	2.642 (1.116–6.252)	0.027		
*CA19-9 (U/ml)*				
< 16.6	Reference			
≥ 16.6	2.234 (1.168–4.273)	0.015		
*SCC (ng/ml)*				
< 16.6	2.052 (1.023–4.113)	0.043	4.795 (2.019–11.388)	< 0.001
≥ 16.6	Reference		Reference	
*NSE (ng/ml)*				
< 14.66	Reference			
≥ 14.66	1.976 (1.051–3.713)	0.034		

*NSCLC*, non-small cell lung cancer; *OR*, odds ratio; *SUV*, standardized uptake value; *SUVmax*, maximum SUV; *SUVpeak*, peak of SUV; *SUVmean*, average of SUV; *MTV*, metabolic tumor volume; *TLG*, total lesion glycolysis; *CEA*, carcinoembryonic antigen; *CYFRA21-1*, cytokeratin 19 fragment; *CA19-9*, carbohydrate antigen 19-9; *SCC*, squamous cell carcinoma antigen; *NSE*, neuron-specific enolase

The results of further multivariate regression analysis revealed that the SUVmax of the mediastinal lymph node (≥ 2.49: OR 7.215, 95% CI 3.326–15.649), SUVpeak of the primary tumor (≥ 2.92: OR 5.717, 95% CI 2.094–15.605), CEA (≥ 3.94 ng/ml: OR 2.467, 95% CI 1.182–5.149), and SCC (< 1.15 ng/ml: OR 4.795, 95% CI 2.019–11.388) were independent significant predictors for metastasis in mediastinal lymph nodes (Table 3).

In the subgroup analysis of lung adenocarcinoma patients, the SUVmax of the mediastinal lymph node (≥ 2.49: OR 8.067, 95% CI 3.193–20.383), SUVpeak of the primary tumor (≥ 2.92: OR 9.219, 95% CI 3.096–27.452) and CA19-9 (≥ 1 6.6 U/ml: OR 3.750, 95% CI 1.485–9.470) were proven to be independent significant predictors for metastasis in mediastinal lymph nodes through multivariate regression analysis.

### Model validation

**Fig. 2:**
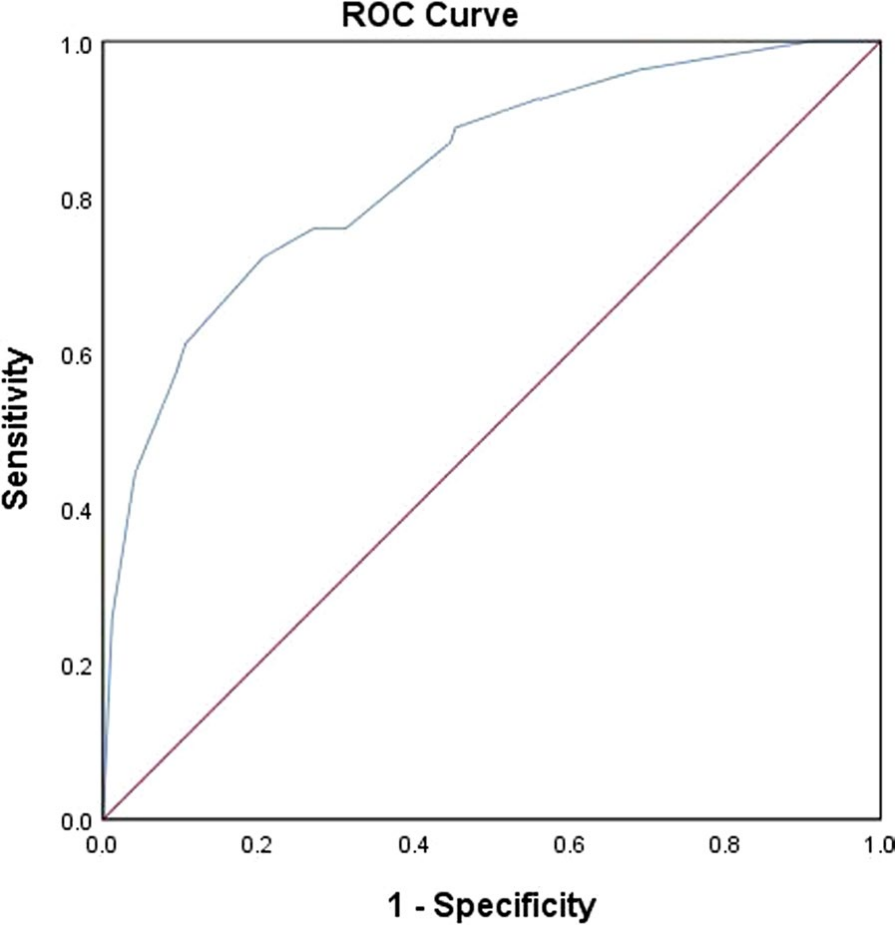
The ROC curve for internal validation of the multivariate prediction model for mediastinal lymph nodes in NSCLC patients: N = 224, AUC was 0.833 (95% CI 0.769–0.896, *p* < 0.001)

**Fig. 3:**
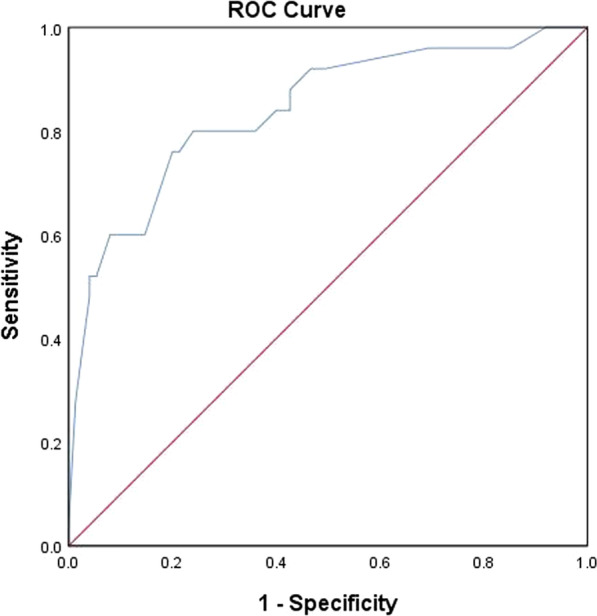
The ROC curve for external validation of the multivariate prediction model for mediastinal lymph nodes in NSCLC patients: N = 100, AUC was 0.811 (95% CI 0.712–0.911, *p* < 0.001)

The AUC based on internal validation of 224 NSCLC patients was 0.833 (95% CI 0.769–0.896, *P* < 0.001). (Fig. 2) The AUC was 0.811 (95% CI 0.712–0.911, *P* < 0.001), in line with external verification from 100 NSCLC patients. (Fig. 3).

**Fig. 4:**
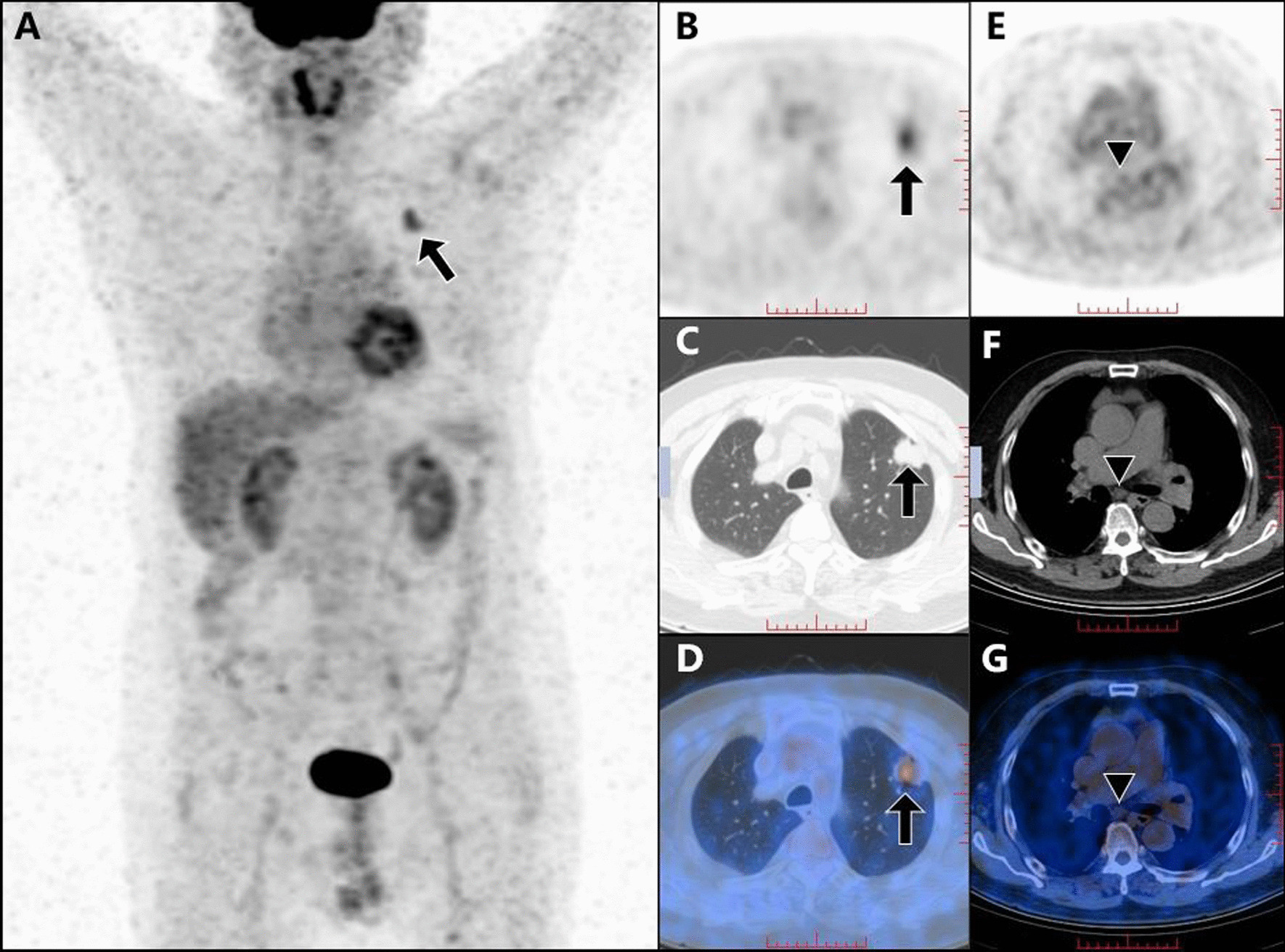
An elderly male (**A–G**) with low-level SUVmax (2.04) of mediastinal lymph node, low-level primary-tumor SUVpeak (3.34), low serum CEA (1.69 ng/ml) and high serum SCC (2.5 ng/ml). The primary tumor, which was confirmed as lung adenocarcinoma through operative pathological assay, is shown in ^18^F-FDG PET/CT maximum intensity projection (MIP) image (**A**), PET (**B**), CT (**C**) and PET/CT (**D**) images (arrows). The mediastinal lymph node in area 7, which was the highest-SUVmax (2.04) node in the mediastinum and proved to be negative by postoperative pathological analysis, was displayed in ^18^F-FDG PET (**E**), CT (**F**) and PET/CT (**G**) images (triangles)

**Fig. 5:**
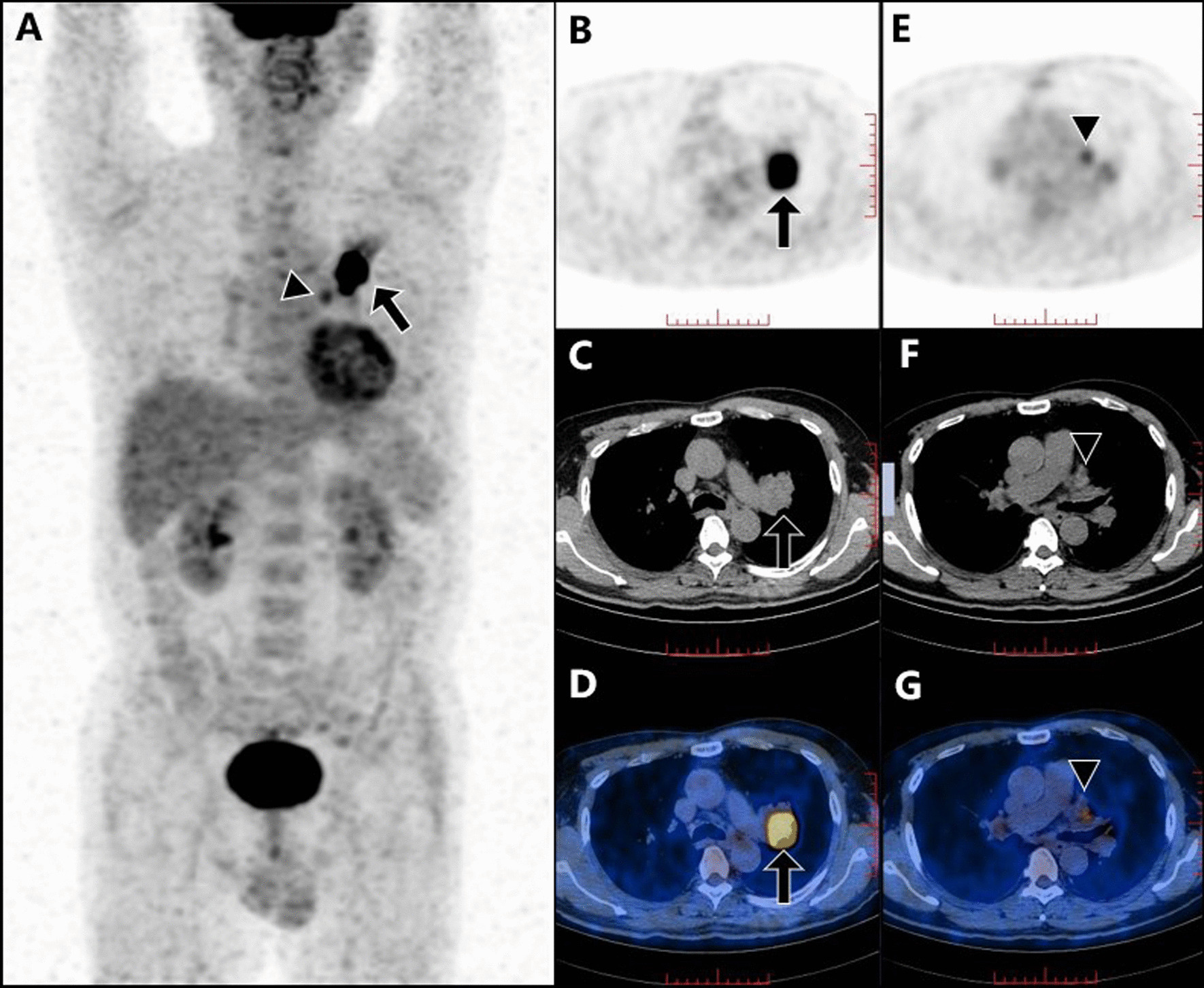
An elderly male (**A–G**) with high-level SUVmax (3.20) of mediastinal lymph node, high-level primary-tumor SUVpeak (9.48), high serum CEA (5.25 ng/ml) and low serum SCC (0.70 ng/ml). The primary tumor, which was confirmed as moderately differentiated squamous cell carcinoma of the lung, is shown in ^18^F-FDG PET/CT maximum intensity projection (MIP) (**A**), PET (**B**), CT (**C**) and PET/CT (**D**) images (arrows). The mediastinal lymph node in area 5, which was the highest SUVmax (3.20) node in the mediastinum and proved to be metastatic by postoperative pathological analysis, was displayed in ^18^F-FDG MIP (**A**), PET (**E**), CT (**F**) and PET/CT (**G**) images (triangle)

Representative cases are shown in Figs. 4 and 5.

## Discussion

It has been reported that SUVs (SUVmax, SUVmean, MTV, TLG, etc.) from ^18^F-FDG PET/CT have potential application value in predicting mediastinal lymph node metastasis in NSCLC patients [7, 11, 12]. However, the results of previous studies suggest that there are differences in the predictive value of these SUVs, and there are also differences in the predictive cutoff value of the same predictive SUV [7, 11]. A meta-analysis pointed out that the semiquantitative SUVs of ^18^F-FDG PET/CT were significantly influenced by the subjects, regions, models, scanner makers, ^18^F-FDG dose and other factors [7]. In view of the importance of mediastinal lymph nodes in treatment decisions, all larger centres should actively make reliable decisions based on their own results and identify the populations in which PET/CT is of most use or potentially little value [7].

The chi-square test and univariate analysis in this study showed that the SUVmax of the mediastinal lymph node and primary-tumor SUVs (SUVmax, suvpeak, SUVmean, MTV and TLG) were all associated with mediastinal lymph node metastasis. The SUVmax of mediastinal lymph nodes and primary tumor SUVpeak were further considered significant and independent predictive features for metastasis of mediastinal lymph nodes through multivariate analysis and subgroup analysis of lung adenocarcinoma.

Peak standardized uptake values (SUVpeak) were the highest SUVmean calculated within a spherical VOI (usually 0.6 cm or 1 cm diameter region) at the site of the most metabolically active tumor manifestation [6]. In other words, SUVpeak is a special SUVmean [6]. Compared with SUVmax, SUVpeak is less disturbed by the statistical fluctuation of radioactive count and can reflect the metabolism of the whole tumor body comprehensively. Compared with SUVmean, SUVpeak has high repeatability for measurement, especially for pulmonary lesions. Because of the small interference of adjacent tissues, SUVpeak’s clinical application is more stable, and the results are more objective and reliable [6].

It has been reported that the predictive value of MTV in the primary tumor is more prominent [11, 12]. However, through multivariate analysis in our study, MTV and TLG of the primary tumor lost their predictive significance and independence. In addition to SUVs, this research collected the maximum diameter of the primary tumor, degree of primary-tumor solidity, serum tumor markers, etc. These parameters were not all considered simultaneously in previous studies [7, 11, 12], and their predictive weight for lymph node metastasis may weaken the significance of MTV or TLG.

Only SUVmax was selected as the semiquantitative parameter of mediastinal lymph nodes in this study. First, SUVpeak, calculated within a spherical VOI (0.6 cm diameter region), was not suitable as an index for lymph nodes because the diameters of many lymph nodes were shorter than 0.6 cm. Then, the tissue structure around mediastinal lymph nodes is complex: large blood vessels, esophagus and other adjacent lymph nodes could cause great interference to the determination of a single lymph node. Therefore, the determination of SUVmean, MTV and TLG, which take into account volumetric information, were susceptible to tissue structure around lymph nodes. SUVmax has higher repeatability [6].

Serum tumor markers related to lung cancer are a series of effective indicators for the tumor burden from the perspective of circulation [14]. A previous study reported the application value of serum CEA in the prediction of metastasis of mediastinal lymph nodes [14], which is consistent with our results of univariate and multivariate analyses. Additionally, our research also discovered that CYFRA21-1, CA19-9, SCC and NSE have different degrees of predictive power in mediastinal lymph node determination. Among them, SCC had independent and significant statistical significance confirmed by multivariate analysis, and CA19-9 was an independent and significant predictor in the subgroup analysis of lung adenocarcinoma. SCC is a specific marker of squamous cell tumors. Our study found that adenocarcinoma may be more prone to mediastinal lymph node metastasis than squamous cell carcinoma, even though this difference was not statistically significant (Tables 1 and 2). Our study also suggested that mediastinal lymph node metastasis was more likely to occur when SCC was lower. Since there were mainly two pathological subtypes (adenocarcinoma and squamous cell carcinoma) in our study, it was speculated that squamous cell carcinoma patients were more unlikely to have mediastinal lymph node metastasis than adenocarcinoma patients, which is in line with the outcome of previous studies [7]. At present, there is no research on the correlation between CA19-9 and mediastinal lymph node metastasis in NSCLC patients. However, it has been reported that CA19-9 can effectively predict the prognosis of NSCLC [15, 16], and mediastinal lymph node metastasis is one of the vital factors for prognosis and survival.

Limitations of this study: first, there might be selective bias because of the retrospective design, especially the sample availability. Second, the sample size was not large enough; in particular, the sample size of patients with squamous cell carcinoma was relatively small. The proportion of adenocarcinoma and squamous cell carcinoma in this study (adenocarcinoma/squamous cell carcinoma = 2.84) was slightly higher than that in the epidemiological report (2.75) [17]. Therefore, the sample validation needs to be further expanded in the follow-up of this study.

## Conclusion

High SUV-derived parameters (SUVmax of mediastinal lymph node and primary-tumor SUVmax, SUVpeak, SUVmean, MTV and TLG) might provide varying degrees of predictive value for mediastinal lymph node metastasis in NSCLC. In particular, the SUVmax of mediastinal lymph nodes and primary tumor SUVpeak could be independently and significantly associated with mediastinal lymph node metastasis in NSCLC and lung adenocarcinoma patients. Internal and external validation confirmed that the pretherapeutic SUVmax of the mediastinal lymph node and primary-tumor SUVpeak combined with serum CEA and SCC can effectively predict mediastinal lymph node metastasis.

## Supplementary Information

**Additional file 1**. **Supplementary Table 1.** Association between other clinical variables and situation of mediastinal lymph nodes in 224 NSCLC patients. **Supplementary Table 2.** Univariate Logistic regression analysis of predictive factors without significance for mediastinal lymph node in NSCLC patients.

## Data Availability

The datasets used and/or analyzed during the current study are available from the corresponding author on reasonable request.
