# Supplementary material for: The value on SUV-derived parameters assessed on 18F-FDG PET/CT for predicting mediastinal lymph node metastasis in non-small cell lung cancer
Source: BMC Med Imaging. 2023 Apr 5;23:49. doi: 10.1186/s12880-023-01004-7 (PMC10077668; doi:10.1186/s12880-023-01004-7)
Supplement: Supplementary file 1 — Additional file 1. Supplementary Table 1. Association between other clinical variables and situation of mediastinal lymph nodes in 224 NSCLC patients. Supplementary Table 2. Univariate Logistic regression analysis of predictive factors without significance for mediastinal lymph node in NSCLC patients. [file 12880_2023_1004_MOESM1_ESM.docx]

| **Supplementary Table 1. Association between other clinical variables and situation of mediastinal lymph nodes in 224 NSCLC patients** | | | | | | |
| --- | --- | --- | --- | --- | --- | --- |
| **Variable** | | **Mediastinal lymph node metastasis** | | **Total** | **Value** | **p value** |
|  |  | **No** | **Yes** |  |  |  |
| Age (years) | |  |  |  | 3.739 ^a^ | 0.053 |
|  | ＜62.5 | 72 (69.9%) | 31(30.1%) | 103 (46.0%) |  |  |
|  | ≥62.5 | 98 (81.0%) | 23 (19.0%) | 121 (54.0%) |  |  |
| Gender | |  |  |  | 0.422 ^a^ | 0.516 |
|  | Male | 106 (77.4%) | 31 (22.6%) | 137 (61.2%) |  |  |
|  | Female | 64 (73.6%) | 23 (26.4%) | 87 (38.8%) |  |  |
| Smoking status | |  |  |  | 0.130 ^a^ | 0.718 |
|  | Never | 96 (75.0%) | 32 (25.0%) | 128 (57.1%) |  |  |
|  | Current or former | 74(77.1%) | 22 (23.1%) | 96 (42.9%) |  |  |
| **Primary tumor position 1** | |  |  |  | 0.034 ^a^ | 0.853 |
|  | Left lung | 78 (76.5%) | 24 (23.5%) | 102 (45.5%) |  |  |
|  | Right lung | 92 (75.4%) | 30 (24.6%) | 122 (54.5%) |  |  |
| **Primary tumor position** 2 | |  |  |  | 6.275 ^b^ | 0.280 |
|  | URL | 41 (69.5%) | 18 (30.5%) | 59 (26.3%) |  |  |
|  | MRL | 10 (71.4%) | 4 (28.6%) | 14 (6.3%) |  |  |
|  | LRL | 43 (87.8%) | 6 (12.2%) | 49 (21.9%) |  |  |
|  | ULL | 43 (74.1%) | 15 (25.9%) | 58 (25.9%) |  |  |
|  | LLL | 27 (77.1%) | 8 (22.9%) | 35 (15.6%) |  |  |
|  | Involving more than 1 lobe | 6 (66.7%) | 3 (33.3%) | 9 (4.0%) |  |  |
| **Primary tumor position** 3 | |  |  |  | 0.123 ^a^ | 0.726 |
|  | Central | 31 (73.8%) | 11 (26.2%) | 42 (18.8%) |  |  |
|  | Peripheral | 139 (76.4%) | 43 (23.6%) | 183 (81.3%) |  |  |
| Pathologic subtype | |  |  |  | 1.700 ^a^ | 0.192 |
|  | ADC | 121 (74.7%) | 41 (25.3%) | 162 (75.0%) |  |  |
|  | SQCC | 45 (83.3%) | 9 (16.7%) | 54 (25%) |  |  |
| *EGFR* mutation | |  |  |  | 1.158 ^a^ | 0.282 |
|  | No | 129 (77.7%) | 37 (22.3%) | 166 (74.1%) |  |  |
|  | Yes | 41 (70.7%) | 17 (29.3%) | 58 (25.9%) |  |  |
| ALK rearrangement | |  |  |  | 0.451 ^c^ | 0.502 |
|  | No | 156 (76.1%) | 49 (23.9%) | 205 (93.6%) |  |  |
|  | Yes | 9 (64.3%) | 5 (35.7%) | 14 (6.4%) |  |  |
| TPA (U/L) | |  |  |  | 2.243 ^a^ | 0.134 |
|  | ＜63.66 | 49 (83.1%) | 10 (16.9%) | 59 (26.3%) |  |  |
|  | ≥63.66 | 121 (73.3%) | 44 (26.7%) | 165 (73.7%) |  |  |
| proGRP (pg/ml) | |  |  |  | 1.594 ^a^ | 0.207 |
|  | ＜34.32 | 46 (82.1%) | 10 (17.9%) | 56 (25.0%) |  |  |
|  | ≥34.32 | 124 (73.8%) | 44 (26.2%) | 168 (75.0%) |  |  |
| Abbreviation: *NSCLC*, non-small cell lung cancer; *URL*, Upper lobe of right lung; *MRL*, Middle lobe of right lung; *LRL*, Lower lobe of right lung; *ULL*, Upper lobe of left lung; *LLL*, Lower lobe of left lung; *FDG*, fluorodeoxyglucose; *ADC*, adenocarcinoma; *SQCC*, squamous cell carcinoma; *EGFR*, epidermal growth factor receptor; *ALK*, anaplastic lymphoma kinase; *TPA*, tissue polypeptide antigen; *proGRP*, precursor of gastrin releasing peptide  ^a^: Pearson chi-square test. ^b^: Likelihood ratio chi-square test. ^c^: Continuity correction chi-square test. | | | | | | |

**Supplementary Table 2. Univariate Logistic regression analysis of predictive factors without significance for mediastinal lymph node in NSCLC patients**

| **Characteristics** | | **Univariate analysis OR (95% CI)** | **P value** |
| --- | --- | --- | --- |
| Age (years) | |  |  |
|  | < 62.5 | 1.835 (0.988-3.408) | 0.055 |
|  | ≥ 62.5 | Reference |  |
| Gender | |  |  |
|  | Male | Reference |  |
|  | Female | 1.229 (0.660-2.289) | 0.516 |
| Smoking | |  |  |
|  | No | 1.121 (0.602-2.088) |  |
|  | Yes | Reference | 0.718 |
| Primary tumor position 1 | |  |  |
|  | Left lung | Reference |  |
|  | Right lung | 1.060 (0.573-1.962) | 0.853 |
| Primary tumor position 3 | |  |  |
|  | Central | 1.147(0.532-2.473) | 0.726 |
|  | Peripheral | Reference |  |
| Primary solid tumor maximum diameter (mm) | |  | 0.171^a^ |
|  | < 32.50 | Reference |  |
|  | ≥ 32.50 | 1.450 (0.720-2.921) | 0.298^b^ |
| Pathologic subtype | |  |  |
|  | ADC | 1.694 (0.762-3.765) | 0.196 |
|  | SQCC | Reference |  |
| EGFR mutation | |  |  |
|  | No | Reference |  |
|  | Yes | 1.446 (0.737-2.834) | 0.283 |
| ALK rearrangement | |  |  |
|  | No | Reference |  |
|  | Yes | 1.769 (0.566-5.527) | 0.327 |
| TPA (U/L) | |  |  |
|  | ＜63.66 | Reference |  |
|  | ≥63.66 | 1.782 (0.831-3.820) | 0.138 |
| proGRP (pg/ml) | |  |  |
|  | ＜34.32 | Reference |  |
|  | ≥34.32 | 1.632 (0.759-3.509) | 0.201 |

Abbreviation: *NSCLC*, non-small cell lung cancer; *OR*, odds ratio; *ADC*, adenocarcinoma; *SQCC*, squamous cell carcinoma; *EGFR*, epidermal growth factor receptor; *ALK*, anaplastic lymphoma kinase; *TPA*, tissue polypeptide antigen; *proGRP*, precursor of gastrin releasing peptide
